# Aberrant spinal mechanical loading stress triggers intervertebral disc degeneration by inducing pyroptosis and nerve ingrowth

**DOI:** 10.1038/s41598-020-80756-6

**Published:** 2021-01-12

**Authors:** Fangda Fu, Ronghua Bao, Sai Yao, Chengcong Zhou, Huan Luo, Zhiguo Zhang, Huihao Zhang, Yan Li, Shuxin Yan, Huan Yu, Weibin Du, Yanping Yang, Hongting Jin, Peijian Tong, Zhi-tao Sun, Ming Yue, Di Chen, Chengliang Wu, Hongfeng Ruan

**Affiliations:** 1 Institute of Orthopaedics and Traumatology, the First Affiliated Hospital of Zhejiang Chinese Medical University, Hangzhou, 310053 Zhejiang China; 2 Hangzhou Fuyang Hospital of TCM Orthopedics and Traumatology, Hangzhou, 311400 Zhejiang China; 3 Department of Pharmacy, the Second Affiliated Hospital, Zhejiang University School of Medicine, Hangzhou, 310009 Zhejiang China; 4 Research Institute of Orthopedics, the Affiliated JiangNan Hospital of Zhejiang Chinese Medical University, Hangzhou, 311200 Zhejiang China; 5 Longhua Hospital, Affiliated to Shanghai University of Traditional Chinese Medicine, Shanghai, 200032 China; 6 Department of Orthopedics, Shenzhen Traditional Chinese Hospital, Guangzhou University of Chinese Medicine, Shenzhen, 518055 China; 7 Department of Physiology, Zhejiang Chinese Medical University, Hangzhou, 310053 China; 8 Research Center for Human Tissues and Organs Degeneration, Shenzhen Institutes of Advanced Technology, Chinese Academy of Sciences, Shenzhen, 518055 China; 9 The First Clinical College, Zhejiang Chinese Medical University, Hangzhou, 310051 Zhejiang China

**Keywords:** Biochemistry, Diseases, Pathogenesis

## Abstract

Aberrant mechanical factor is one of the etiologies of the intervertebral disc (IVD) degeneration (IVDD). However, the exact molecular mechanism of spinal mechanical loading stress-induced IVDD has yet to be elucidated due to a lack of an ideal and stable IVDD animal model. The present study aimed to establish a stable IVDD mouse model and evaluated the effect of aberrant spinal mechanical loading on the pathogenesis of IVDD. Eight-week-old male mice were treated with lumbar spine instability (LSI) surgery to induce IVDD. The progression of IVDD was evaluated by μCT and Safranin O/Fast green staining analysis. The metabolism of extracellular matrix, ingrowth of sensory nerves, pyroptosis in IVDs tissues were determined by immunohistological or real-time PCR analysis. The apoptosis of IVD cells was tested by TUNEL assay. IVDD modeling was successfully produced by LSI surgery, with substantial reductions in IVD height, BS/TV, Tb.N. and lower IVD score. LSI administration led to the histologic change of disc degeneration, disruption of the matrix metabolism, promotion of apoptosis of IVD cells and invasion of sensory nerves into annulus fibrosus, as well as induction of pyroptosis. Moreover, LSI surgery activated Wnt signaling in IVD tissues. Mechanical instability caused by LSI surgery accelerates the disc matrix degradation, nerve invasion, pyroptosis, and eventually lead to IVDD, which provided an alternative mouse IVDD model.

## Introduction

Low back pain (LBP) is a highly prevalent disabling musculoskeletal disease, affecting as much as 84% of the population worldwide at some point in their lifetime^1^. Intervertebral disc (IVD) degeneration (IVDD) is clinically associated with LBP and recognized as the main pathogenic factor for LBP^2,3^. Previous studies on tissue mechanics showed that persistent aberrant mechanical stress generated by body weight and muscle force is one of the common causes of disc degeneration and associated LBP, and there is a positive relationship between changes in mechanical properties and abnormal changes of IVD structure and composition^4,5^. However, due to a lack of an ideal animal model of persistent instability of IVDD, the exact molecular biological mechanisms by which aberrant spinal mechanical loading initiates and promotes IVDD has not been fully elucidated.

IVDs are the major cartilaginous joints of the vertebral column, contributing about 1/3 of spinal length^6^. Each IVD consists of the central nucleus pulposus (NP) surrounded by lamellated collagenous ring, the annulus fibrosus (AF), and cartilage endplates (EP) comprises of the superior and inferior boundaries of the IVD separating the disc from the vertebrae. On the inner border of the AF, the fibers form a tissue mainly composed of type II collagen (Col2). Constrained by the tight AF, NP is critical water-retaining tissue, producing Aggrecan and other proteoglycans. The complex structural features of IVDs enable to absorb and disperse loads from physical activities and other body parts, as well as maintenance of disc height^7^. Aberrant changes in extracellular matrix (ECM) composition are key to the development of IVDD. Mechanical test on cadaveric lumbar motion segments suggested that complex mechanical loading simulating typical activities in vivo could damage IVDs, including gross structural disruption as well as cell-mediated matrix composition changes, and eventually result in IVDD^8^. However, the mechanical factors driving IVD cell transition are still unclear, particularly how the mechanical load influences cell signaling.

Recent studies have postulated that both chronic inflammation and innervation are both key contributors to LBP^9–11^. Pyroptosis, also known as cell inflammatory necrosis, is a newly discovered programmed cell death that manifests as cells continue to swell until the cell membrane ruptures, resulting in the release of cell contents and activation of a strong inflammatory response^12^. Pyroptosis is mediated by the formation of inflammasome complexes, which are cytosolic heptameric oligomers composed of the nucleotide-binding domain and leucine-rich repeat (NLR) pattern recognition receptors. The best-characterized NLR, NLRP3, is a redox-sensitive cytosolic sensor, which triggers the docking and activation of pro-Caspase-1, thus cleaving pro-IL-1β into 17-kDa mature active forms, IL-1β^13,14^. Nevertheless, during the development of IVDD, the role of pyroptosis is remains obscure. Moreover, a previous clinical study found that nerve ingrowth is abundant and maximal in degenerate IVDs of patients expressing pain^15^. Similarly, a puncture-induced disc herniation in the rodent also showed that puncture-induced damage to IVDs resulted in pinched nerves, which is potential pathogenesis of pain in the lower back and legs^16^. However, the underlying mechanism has not yet entirely understood.

Although the biological mechanisms controlling mechanical transduction has not been completely defined, considerable research have revealed that Wnt/β-catenin signaling plays an essential role in mechanically induced signal transduction, thus participating in the regulation of normal disc metabolism and diseased states^17–19^. Both canines with an age-related propensity for degeneration and IVDD patients have up-regulated levels of β-catenin^5,20^. The analysis of Wnt signaling using mouse models has been indispensable in further elucidating the development of IVD and the occurrence of disc diseases^21^. Mice haploinsufficient for *β-catenin* in osteocytes and/or late-stage osteoblasts showed no measurable anabolic response to mechanical stimulation *in vivo*^22^. The activation of Wnt signaling allows nuclear-translocated β-catenin to combine with the transcription factors TCF proteins and activates expression of target genes, such as *Dkk1* and *Lef1*^23,24^. Specifically blocking Wnt/β-catenin signaling pathway by Dkk-1 could reverse TNF-α induced disc degeneration and protect the normal metabolism of intervertebral disc tissue^25^.

To the best of our knowledge, the most common IVDD model was created by needle puncturing in IVDs, which is difficult to simulate the non-injured degeneration of the human IVDD^26^. Therefore, the purpose of this study was to construct and characterize an aberrant spinal mechanical loading induced IVDD model which can help to gain a better understanding of the pathophysiologic events during mechanical instability-induced pathogenesis of IVDD.

## Results

### Aberrant mechanical stress leads to IVDD

**Figure 1:**
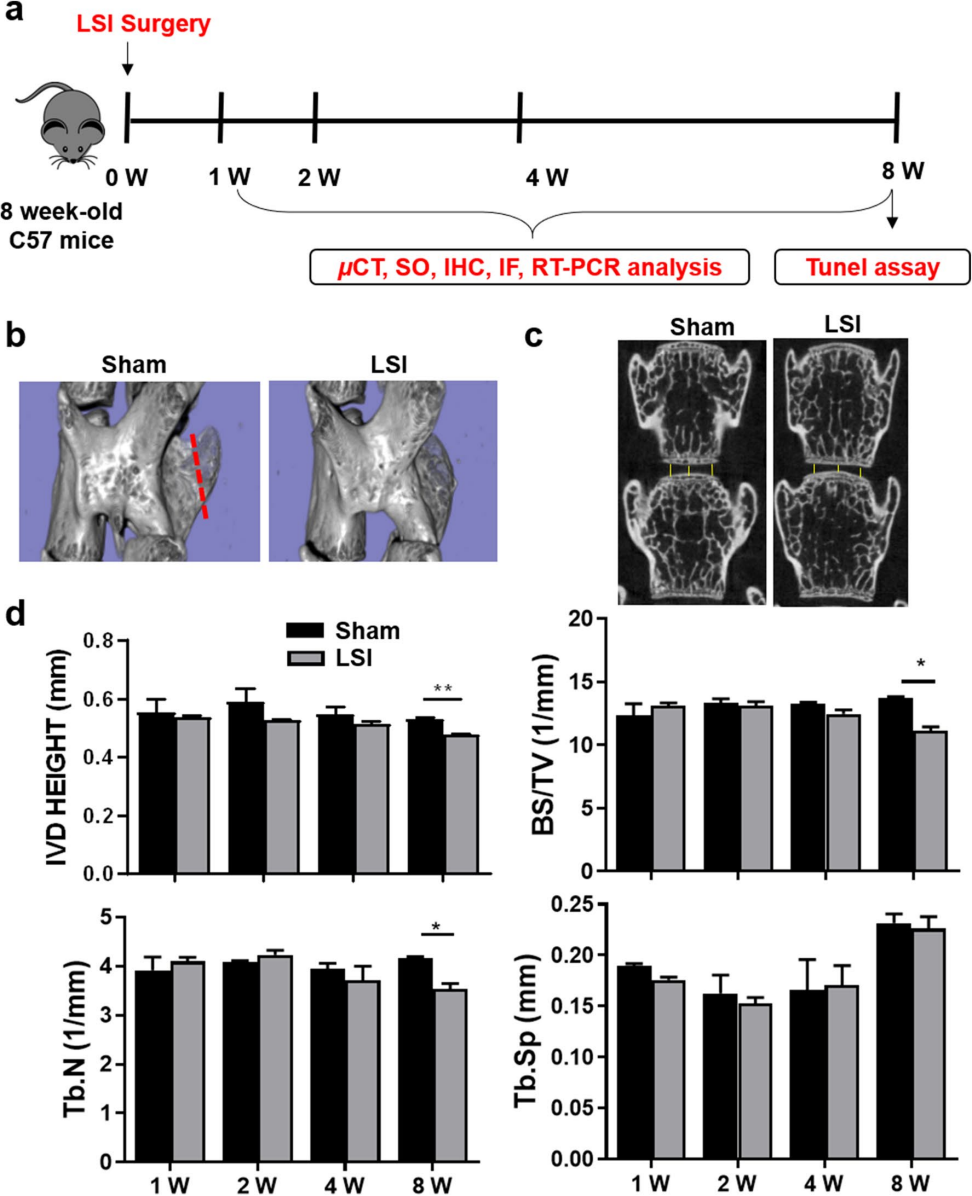
Aberrant mechanical stress reduces the disc height. (**a**) Study design of the project. (**b**) Lumbar spine instability (LSI) mouse model. Mouse L3-L5 spinous processes were resected along with the supraspinous and interspinous ligaments (red dotted line in Sham group) to induce instability of lumbar spine. (**c**) Coronal μCT of the lumbar spine. The images were obtained by using CTVol v2.0 software. The yellow line represents the distance between the middle to middle or side to side of the adjacent vertebra. (**d**) Intervertebral L4-L5 disc height and bone histomorphometry (BS/TV, Tb.N, Tb.Sp) of L5 vertebral body were analyzed at 1-, 2-, 4-, and 8-week (W) post-surgery. Data are shown as mean ± sem. *P < 0.05, **P < 0.01 compared to Sham group.

To simulate the occurrence of IVDD induced by aberrant mechanical stress, we constructed a mouse lumbar spine instability (LSI) model by removing spinous processes, posterior supraspinous and interspinous ligaments, so as to construct a mouse IVDD model (Fig. 1a,b). Since the IVD height of mice generally increases from birth to 1-month old and then remains stable before decreasing around 4-month old, the loss of IVD height is generally considered as a surrogate predictor of IVDD^27^. We firstly evaluated the disc height of the L4-L5 IVD at 1-, 2-, 4- and 8-week after LSI surgery by micro-computed tomography (μCT) analysis. We found that the disc height of LSI mice was significantly decreased 8-week post-surgery (Fig. 1c,d). Moreover, bone histomorphometry analysis showed that LSI treatment resulted in significant reductions both in BS/TV and Tb.N (Fig. 1d).

### Aberrant mechanical stress disruptes the morphology of IVD tissues

**Figure 2:**
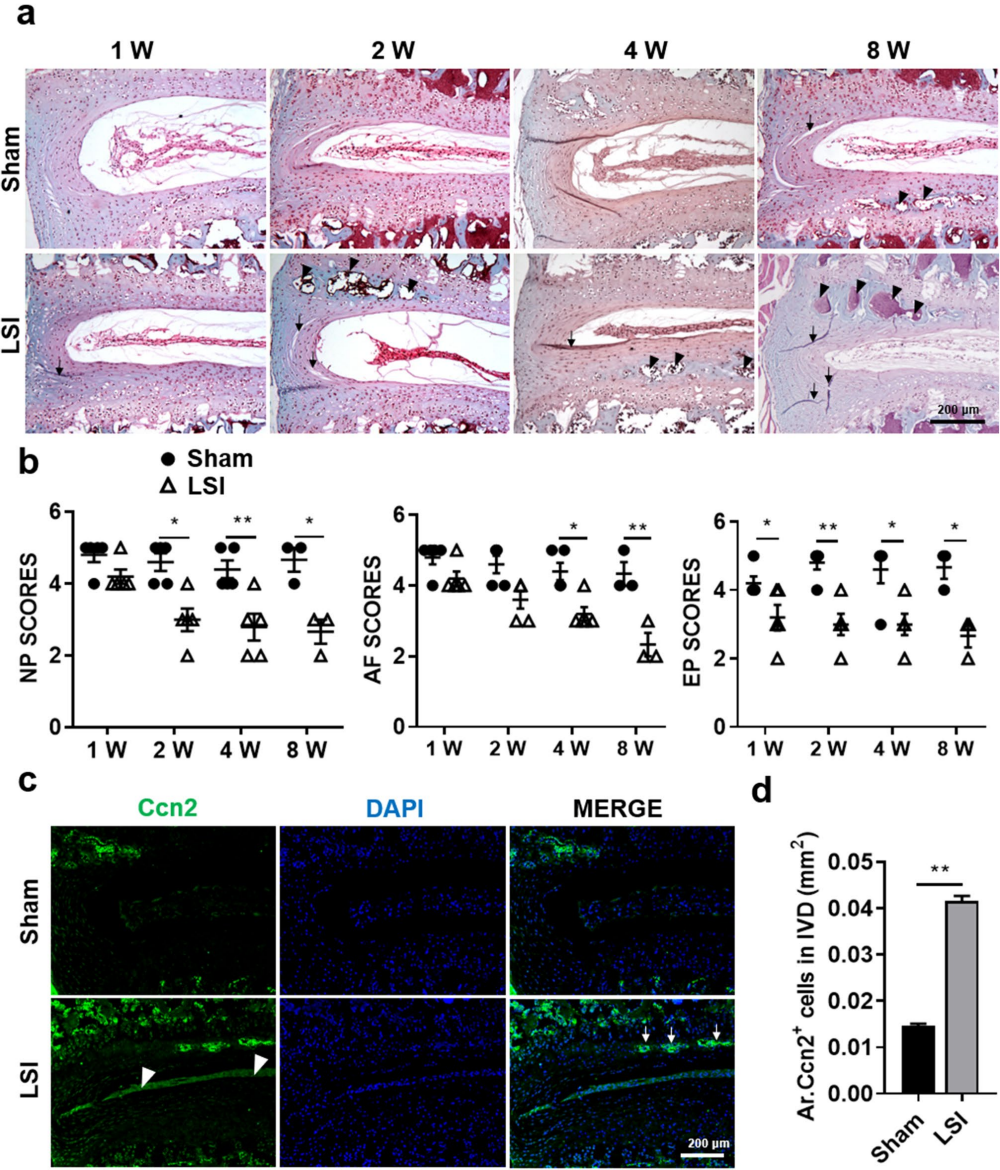
Aberrant mechanical stress disruptes the morphology of the intervertebral disc and activates Ccn2 expression. (**a**) Representative Safranin O staining images of the IVD sections showing the changes of IVD cells in Sham and LSI mice at 1-, 2-, 4- and 8-W post-surgery. Black arrows indicate tearing in AF. Black arrow heads indicate ectopic bone formation in EP. (**b**) Evaluation of IVD degeneration by NP, AF and EP score. (**c**) Immunofluorescence staining for Ccn2 expression (green) at 4-W post-surgery. DAPI stains nuclei blue. White arrows indicate high expression of Ccn2 in the ectopic bone formation zone of EP. White arrowheads indicate high expression of Ccn2 in NP tissues. (**d**) Quantification of Ccn2-positive cells in IVD of (**c**). The averages of integrated optical density in interest region was calculated by Image-Pro Plus software version 6.0 (https://www.mediacy.com). Data are shown as mean ± sem. Three independent experiments performed in triplicate. *P < 0.05, **P < 0.01 compared to Sham group.

To investigate whether LSI surgery could affect the structure and composition of IVDs, we examined the histological changes of IVD between L4-L5 by Safranin O/Fast green staining. Histological stain analysis results showed that IVDs of Sham mice had well-organized tissue structures, including large vacuoles with sufficient matrix content in NP, arranged regularly AF tissues without any tearing, and no ectopic bone formation in EP. Meanwhile, LSI surgery treatment led to a significant decrease in height of discs, vacuole sizes in NP, ectopic bone formation in EP, accompanied by fissures and folds in the interlamellar of AF tissues (Fig. 2a). The degeneration of IVD was further assessed by histological score system established by Norcross et al.^28^. The score results suggested that NP and EP had significant decreases since 2-week after surgery, followed by decreases of AF scores 4- and 8-weeks after surgery (Fig. 2b). Consistent with these results, the levels of Ccn2, a factor that regulates matrix protein synthesis in IVDs and an indicator of disc degeneration^27^, were about threefold higher in LSI mice than in Sham mice (Fig. 2c,d).

### Abnormal mechanical stress damages matrix composition of IVD tissues

**Figure 3:**
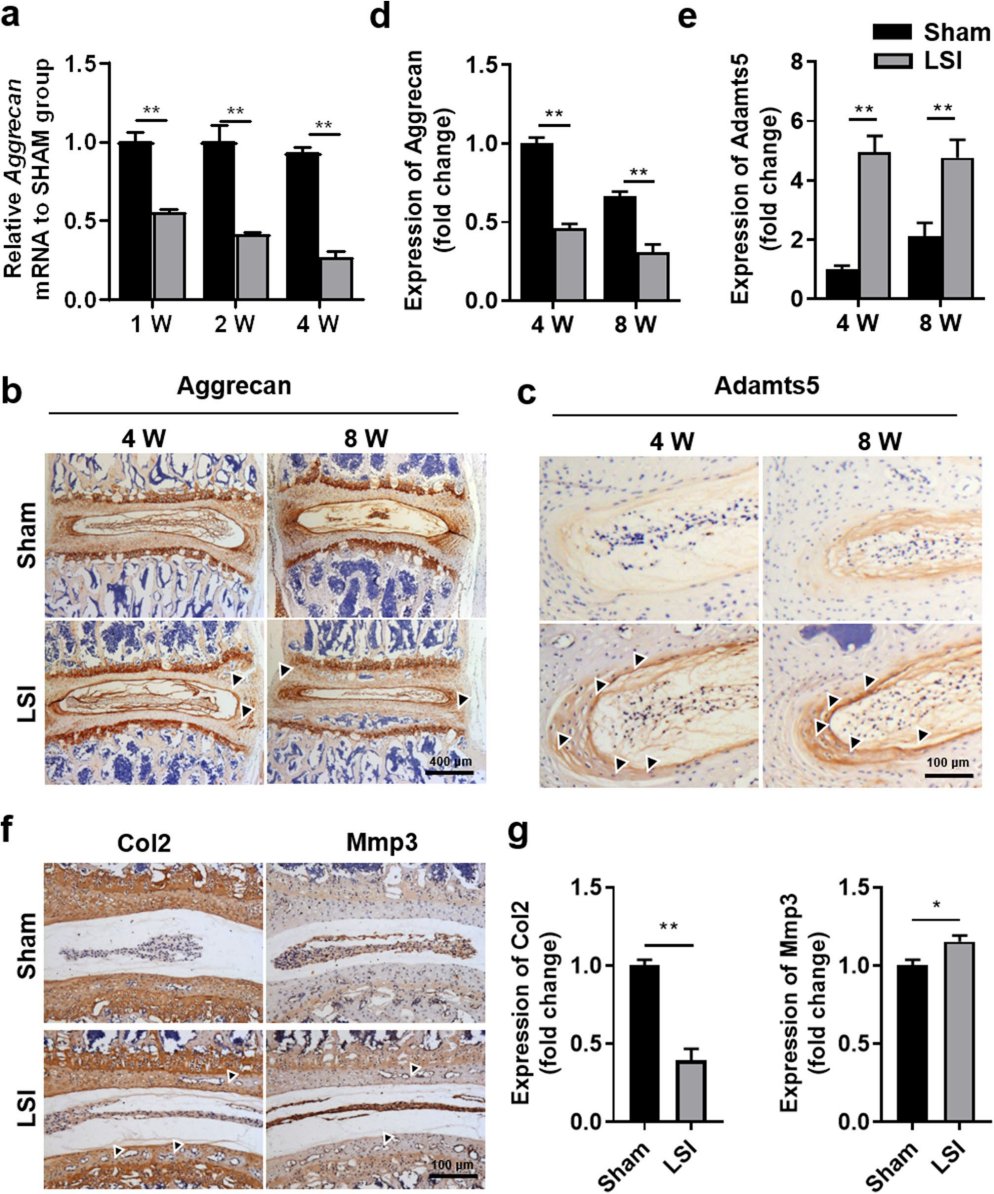
Aberrant mechanical stress resulted in matrix composition changes in the intervertebral disc. (**a**) Immunostaining for Aggrecan in the IVD (brown) at 4-, 8-W post-surgery. Hematoxylin stains nuclei purple. Black arrowheads indicate low expression of Aggrecan in AF. (**b**) Immunostaining for Adamts5 in the AF (brown) at 4-, 8-W post-surgery. Black arrowheads indicate the high expression of Aggrecan in AF. (**c**) qPCR analysis for Aggrecan mRNA in the IVD at 1-, 2- and 4-W post-surgery. (**d**) Quantification of Aggrecan expression in IVD of (**a**). (**e**) Quantification of Adamts5 expression in IVD of (**b**). (**f**) Immunostaining for Col2 and MMP3 (brown) at 8-W post-surgery. Black arrowheads indicate low expression of Col2 in EP or high expression of Mmp3 in EP. (**g**) Quantification of Col2 and Mmp3 expression in EP region of (**e**). The averages of integrated optical density in interest region was calculated by Image-Pro Plus software version 6.0 (https://www.mediacy.com). Data are shown as mean ± sem. Three independent experiments were performed in triplicate. *P < 0.05, **P < 0.01 compared to Sham group.

Generally, stable matrix content maintains the physiological function of the IVD. Col2 and aggrecan are the principal components present in disc tissue, while Mmp3 and Adamts5 are known as key degradases for degrading Col2 and Aggrecan, respectively^20^. To determine the influence of mechanical instability on matrix metabolism, we first quantified the *Aggrecan* mRNA levels in IVD tissues 1-, 2-, 4-week post-surgery. The qRT-PCR results showed that LSI administration resulted in a significant decrease of *Aggrecan* mRNA levels since 1-week post-surgery (Fig. 3a). Then, we examined protein levels of Aggrecan, Col-II and corresponding matrix-degrading enzymes (Adamts5 and Mmp3, respectively) by immunohistochemistry (IHC) analysis. As shown in Fig. 3b–e, LSI mice exhibited a 54% down-regulation of Aggrecan and threefold upregulation of Adamts5 in the whole disc. Similarly, the expression of Col2 and Mmp3 in the EP region showed approximately the same trend as that of Aggrecan and Adamts5, respectively (Fig. 3f–g).

### Aberrant mechanical stress induces sensory nerve invasion in AF

**Figure 4:**
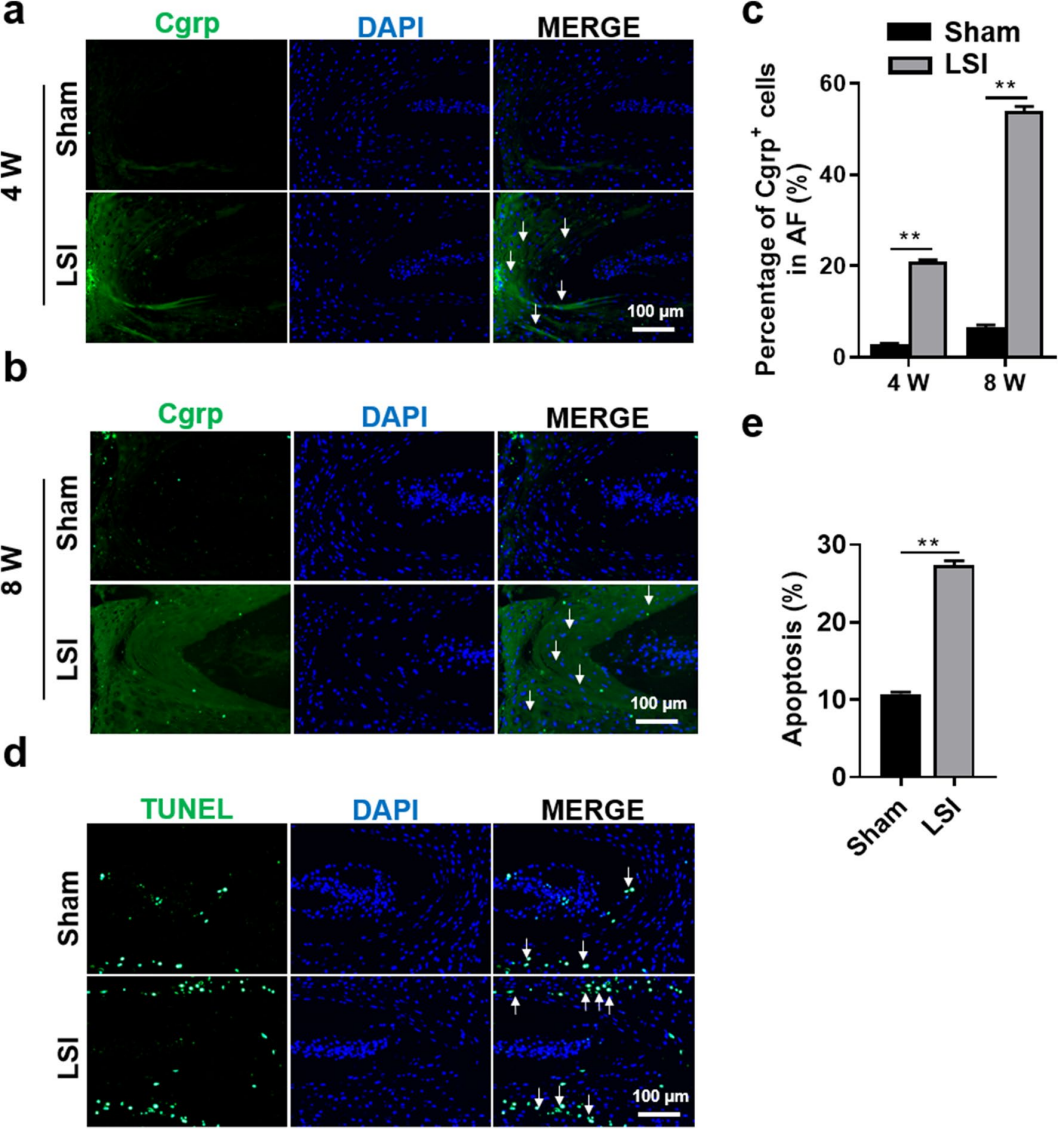
Aberrant mechanical stress stimulated sensory nerve invasion into IVD tissues and deteriorates apoptosis of IVD cells. (**a**, **b**) Immunofluorescence staining for Cgrp expression (green) at 4- and 8-W post-surgery. DAPI stains nuclei blue. White arrows indicate high expression of Cgrp in AF. (**c**) Quantification of Cgrp-positive cells in AF region of (**a**, **b**). (**d**) Apoptosis images of the IVD sections. DAPI stains nuclei blue. White arrows indicate apoptosis in EP cells. (**e**) Quantification of apoptosis rate in (**d**). Data are shown as mean ± sem. Three independent experiments were performed in triplicate. *P < 0.05, **P < 0.01 compared to Sham group.

IVDD is thought to be the cause of LBP partially due to sensory nerve ingrowth into the degenerated IVD^29^. Evidence from animal models and human studies revealed that sensory innervation of lumbar IVD and sensory nerve ingrowth into the inner layer of IVD caused painful conditions^30–32^. To explore the distribution of sensory nerves after IVD modeling, we detected the expression of its specific marker (Cgrp) by immunofluorescent (IF) analysis. The IF results showed that LSI surgery significantly increased the number of Cgrp-positive cells in AF region (Fig. 4a–c).

### Aberrant mechanical stress aggravates apoptosis in IVD

Apoptosis, a process of cellular suicide, also plays a crucial role in IVD degeneration^33^. Then we investigated the effects of LSI surgery on apoptosis of IVD cells by TUNEL assay. We found that the rate of apoptosis in the LSI mice was significantly increased by about 2 times (Fig. 4d,e), especially in the ectopic bone formation zone of EP. These results indicated that aberrant mechanical stress strongly induced apoptosis of IVD cells.

### Aberrant mechanical stress promotes pyroptosis of IVD

**Figure 5:**
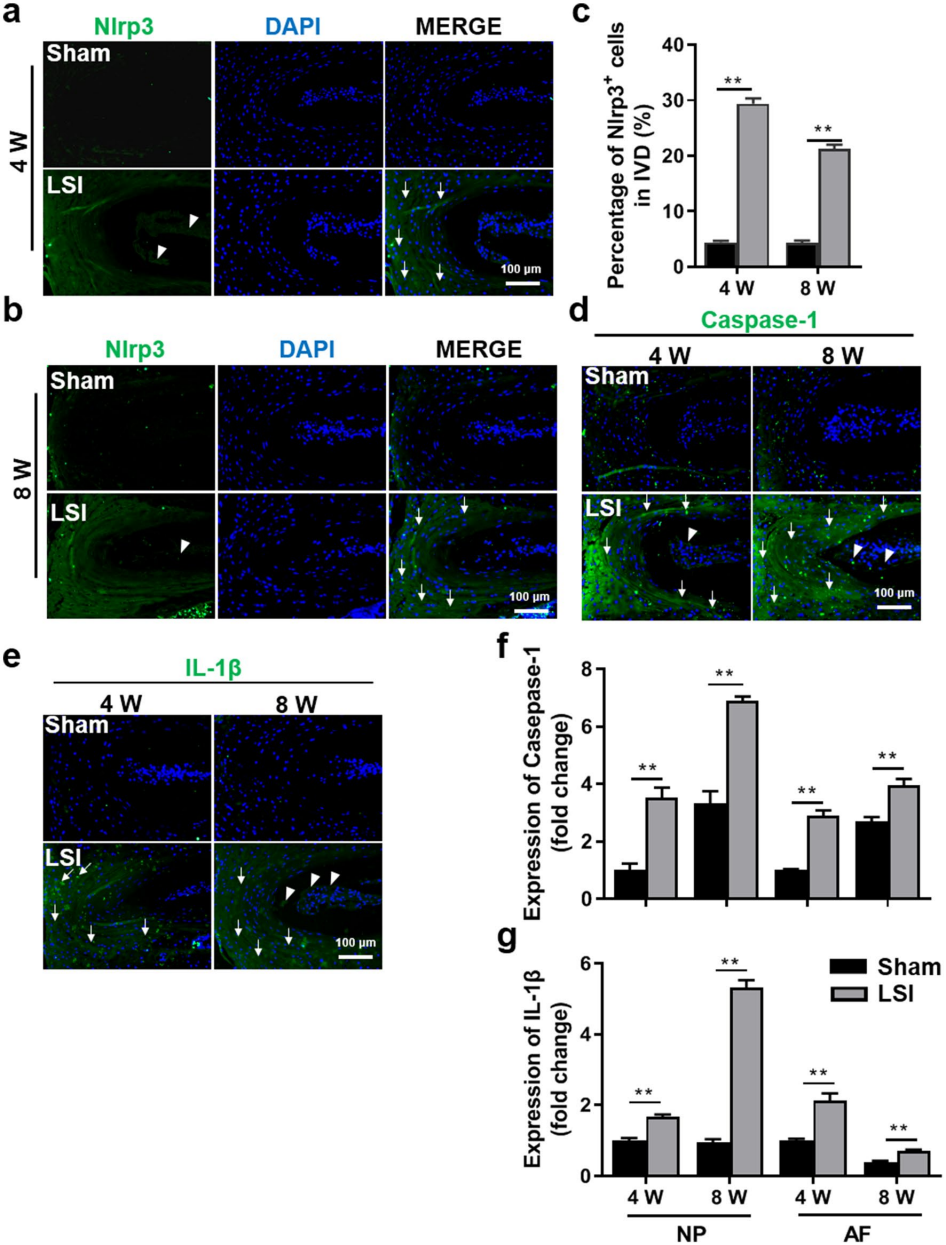
Aberrant mechanical stress-induced pyroptosis in IVD tissues. (**a**, **b**) Immunofluorescence staining for Nlrp3 expression (green) at 4- and 8-W post-surgery. DAPI stains nuclei blue. White arrowheads indicate high expression of Nlrp3 in NP; White arrows indicate high expression of Nlrp3 in AF. (**c**) Quantification of Nlrp3-positive cells in IVD of (**a**, **b**). (**d**, **e**) Immunofluorescence for Caspase-1 (**d**) and IL-1β (**e**) in the IVD (green) at 4- and 8-W post-surgery. DAPI stains nuclei blue. White arrowheads indicate a high expression of Caspase-1 in NP. White arrows indicate a high expression of Caspase-1 and IL-1β in AF. (**f**, **g**) Quantification of Caspase-1 and IL-1β expression in NP or AF region of (**d**, **e**). The averages of integrated optical density in interest region was calculated by Image-Pro Plus software version 6.0 (https://www.mediacy.com). Data are shown as mean ± sem. Three independent experiments were performed in triplicate. *P < 0.05, **P < 0.01 compared to Sham group.

Since chronic inflammation is another key component of painful IVDs^9,10^, we subsequently determined the effect of LSI surgery on the induction of pyroptosis in IVD tissues. IF results of Nlrp3, Caspase-1 and IL-1β, the key proteins of NLRP3 inflammasome signaling, demonstrated that LSI administration induced a significantly increased expression of Nlrp3 in AF and ectopic bone formation zone in EP (Fig. 5a–c). Similarly, LSI treatment resulted in a significant increase of Caspase-1 and IL-1β expression both in NP and AF of LSI mice (Fig. 5d–g).

### Aberrant mechanical stress activates Wnt/β-catenin signaling pathway of IVD tissues

**Figure 6:**
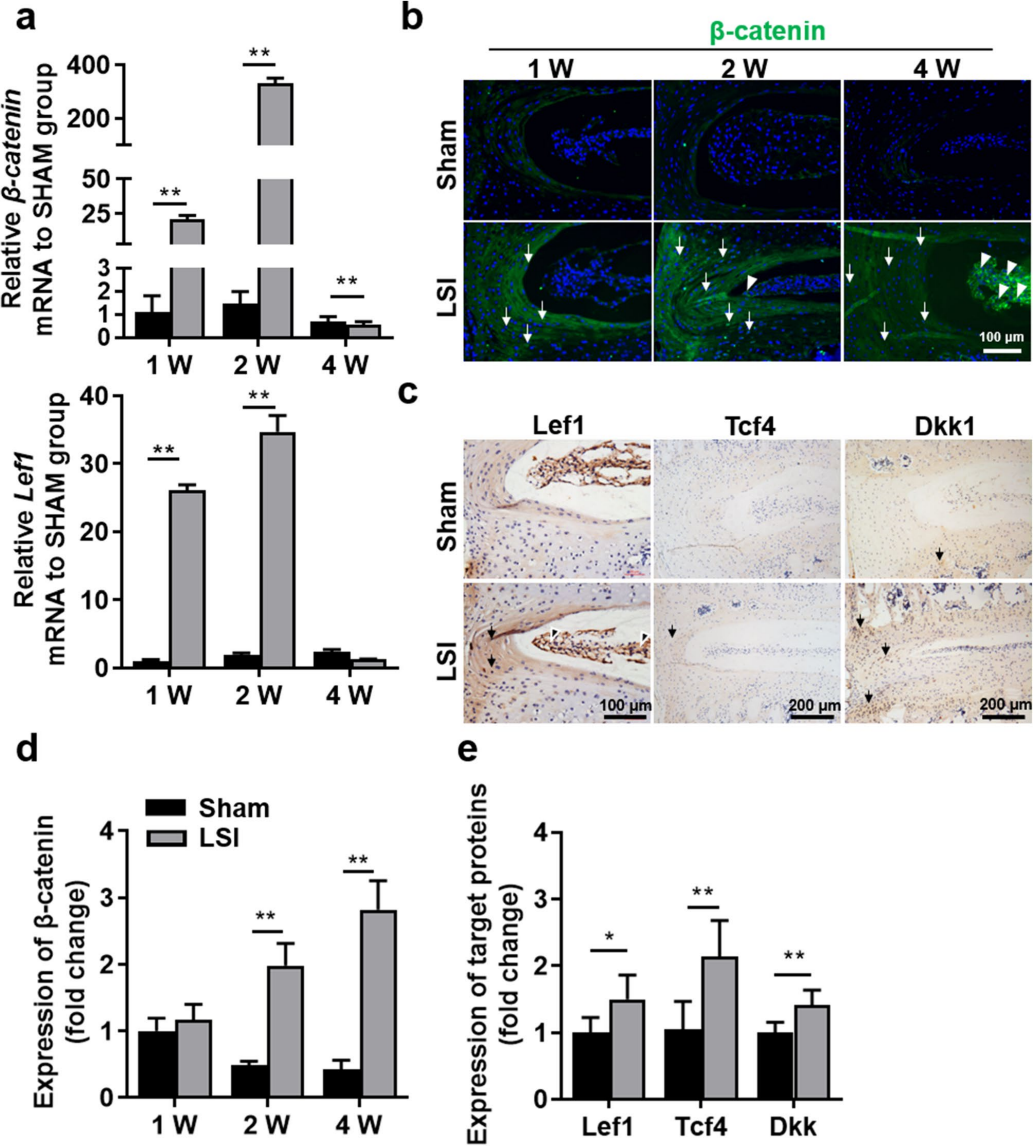
Aberrant mechanical stress activated Wnt/β-catenin signaling pathway of IVD tissues. (**a**) qPCR analysis for *β-catenin*, *Lef1* in the IVD at 1-, 2- and 4-W post-surgery. (**b**) Immunofluorescence for β-catenin in the IVD tissues at 1-, 2- and 4-W post-surgery (green). DAPI stains nuclei blue. White arrowheads indicate high expression of β-catenin in NP. White arrows indicate high expression of β-catenin in AF. (**c**) Immunostaining for Lef1 Tcf4, and Dkk1 in the IVD at 4-W post-surgery (brown). Hematoxylin stains nuclei purple. Black arrows indicate high expression of Lef1, Tcf4 and Dkk1 in AF. (**d**) Quantification of β-catenin expression in IVD of (**b**). (**e**) Quantification of Tcf4, Lef1 and Dkk1 expression in IVD of (**c**). The averages of integrated optical density in interest region was calculated by Image-Pro Plus software version 6.0 (https://www.mediacy.com). Data are shown as mean ± sem. Three independent experiments were performed in triplicate. *P < 0.05, **P < 0.01 compared to Sham group.

Our previous study showed that β-catenin protein was up-regulated in disc tissues from patients with IVDD^20^. In addition, activation of Wnt/β-catenin signaling was also involved in pyroptosis and neuropathic pain^34^. To further explored the effect of LSI treatment on Wnt/β-catenin signaling, the expression levels of *β-catenin* and *Lef1* mRNA in IVD tissues were quantified by qRT-PCR analysis. qRT-PCR results showed that *β-catenin* and *Lef1* mRNA levels were significantly increased in IVDs of LSI mice since 1-week post-surgery (Fig. 6a). Then we further examined protein levels of β-catenin and target proteins of Wnt signaling, including Lef1, Tcf4, and Dkk1. As expected, the IF results of β-catenin revealed that LSI surgery significantly increased the expression of β-catenin in NP and AF (Fig. 6b,d). And the IHC results of Tcf4, Lef1 and Dkk1 showed a similar pattern to β-catenin (Fig. 6c,e). These findings indicate that LSI treatment might promote pyroptosis and nerve ingrowth of IVD tissue by activating the Wnt signaling.

## Discussion

**Figure 7:**
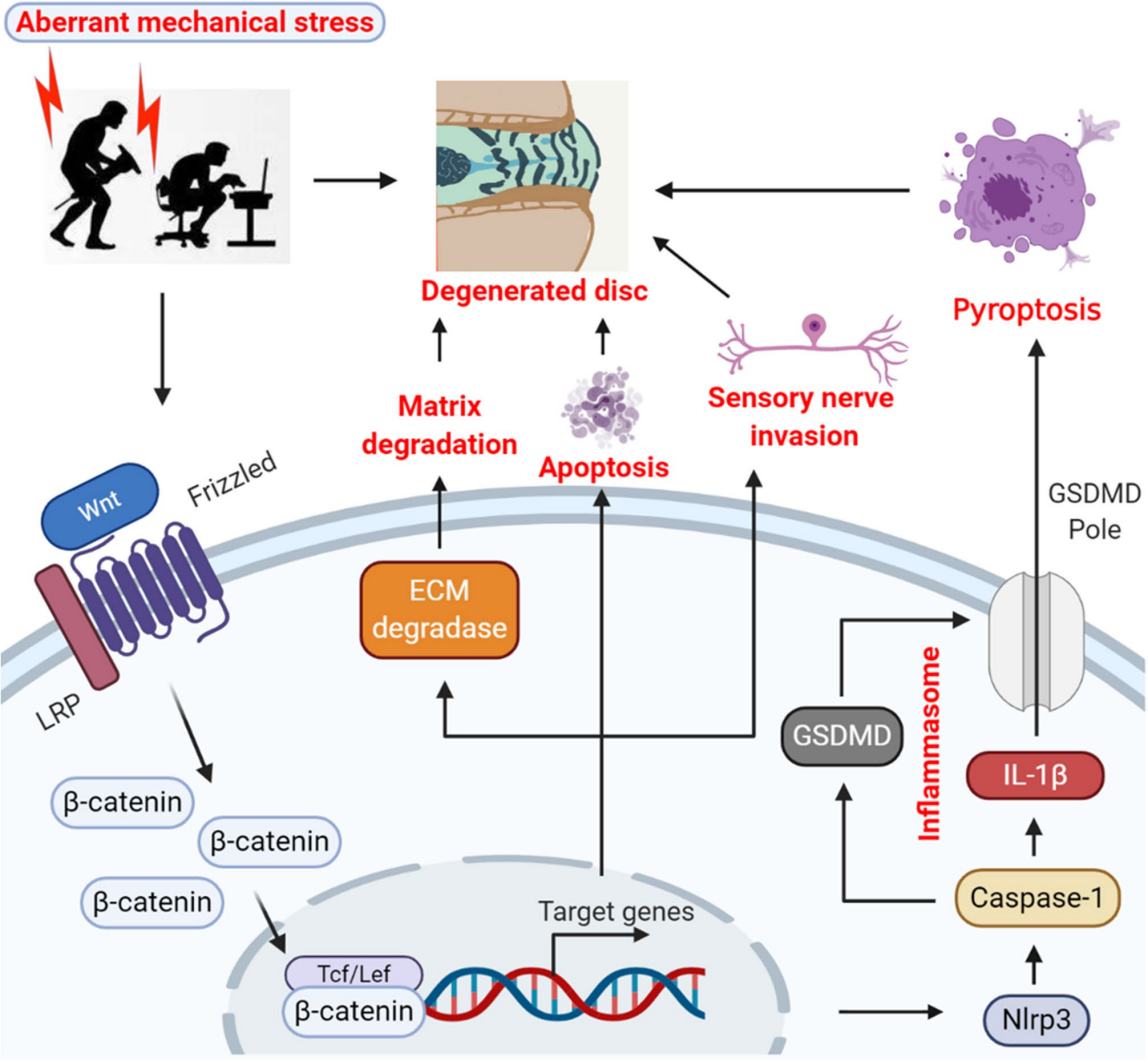
Working model for aberrant mechanical stress-induced IVDD progression by inducing pyroptosis and nerve ingrowth. The image was created by using the biorender software (https://biorender.com/).

Multiple clinical investigations have showed that congenital malformations of spine (including scoliosis, kyphosis, spina bifida, spondylolysis and Klippel Feil syndrome)^35,36^, accidental back injury or ligament injury^37–39^, occupational exposure (such as crane and car drivers, weightlifters, etc.)^40–42^ could induce aberrant mechanical loading of lumbar spine, and finally lead to IVDD. Moreover, decompensated changes in lumbar spine structure result from abnormal mechanical environment, such as proliferation of facet joints^43^, formation of osteophytes^44^, hypertrophy of ligamentum flavum^45^ or calcification of longitudinal ligament^46,47^, will fail to resist aberrant mechanical loading and eventually accelerate the process of IVDD in patients. Since there is no desirable model to mimic the clinical development process of aberrant mechanical loading induced-IVDD more accurately, the underlying molecular events of IVDD pathogenesis remains largely elusive. In this study, we constructed a reproducible mouse IVDD modeling with direct in vivo evidence of medical iconographic and morphological changes in the IVD after loading axial anomalous stress. LSI surgery could promote the progression of IVD degeneration, including notable decreases in IVD height and histological score, ectopic new bone formation in EP, folds and tears in AF, reduction of the vacuole volume in NP, acceleration of ECM degradation, innervation into AF, and induction of pyroptosis after IVDD modeling. In addition, LSI treatment resulted in the activation of Wnt/β-catenin signaling in IVDD mice, which might be responsible for the above changes (Fig. 7). Taken together, we developed a new IVDD model that could help to improve our understanding of the biological events in aberrant spinal axial mechanical loading-induced human IVDD, and presented potential targets for the clinical treatment of IVDD and LBP.

The β-catenin adhesion complex is one of the central components of the cell–cell adhesion junctions, transmitting mechanical stress from cell to cell. Mechanical stress could upregulate β-catenin protein^48–50^, which was also significantly elevated in IVD samples obtained from patients with disc degeneration^51,52^. To develop a mouse model to mimic human IVDD, our previous study has generated an inducible *β-catenin* conditional activation (cAct) mice by breeding *β-catenin*^*fx(Ex3)/fx(Ex3)*^ mice with *Col2a1-CreER*^*T2*^ transgenic mice, according to the up-regulation of β-catenin in disc tissue of IVDD patients. Although these mice have multiple features that resemble some of the features of human IVDD, NP cells were not targeted by *Col2a1-CreER*^*T2*^ transgenic mice and the severely disorganized defect seen in NP tissues may result from the disruption of nutrient and solute supplies to this region and structural changes in AF tissues after the loss of the growth plate cartilage in *β-catenin cAct* mice^20^. Therefore, the phenotype of these mice may only partially resemble the feature of human IVDD. In the present study, we found that LSI surgery significantly increased β-catenin and its target proteins both in NP and AF tissues, which provided an alternative method for simulation of human IVDD.

Clinical evidence has reported that IVD inflammation and axonal growth of afferent fibers innervating the disc are main factors of discogenic LBP, and IL-1β is a pain-related molecule, which is significantly elevated in painful human IVD^53^. The latest research indicated that pyroptosis activation in IVD was likely responsible for the inflammatory pathology of IVDD^54^. And mechanical stress could drive inflammatory responses associated with IVDD and LBP *in vitro*^55^. The important finding in our study is that the pyroptosis was significantly facilitated in outer AF and ectopic bone formation zone inside EP after LSI surgery treatment. The increase of IL-1β induced by pyroptosis, a main inflammatory factor, will further inhibit ECM anabolism and promotes its catabolism in IVDs, as well as the boost of apoptosis in IVD cells.

Besides, the onset of discogenic pain is characterized by ingrowth of nerve fibers into an otherwise aneural tissues^10^. Whether mechanical stress could directly promote nerve ingrowth in AF is an under-investigated aspect. Navone et al.found that mechanical stress could affect nerve proliferation by changing the disc microenvironment^56^. Impaired AF could lead to the loss of focal proteoglycan, and leave a matrix favorable for nerve ingrowth^57^. In this study, we found that IVDD mice exhibited a significant increase in the number of Cgrp-positive sensory nerves both in outer and inner AF. This finding is very similar to the phenomenon in clinical patients, that is, IVDD patients had more nociceptive sensory innervation in AF, which is an important origin of LBP in IVDD patients. In addition, a large number of cytokines released by pyroptosis cells also stimulate sensory nerves, which further leading to more severe LBP. Therefore, anti-pyroptosis and anti-innervation may serve as potential therapeutic strategies for IVDD disease and LBP.

Previous research has reported that sustained mechanical load could activate Wnt/β-catenin signaling in the development of osteoarthritis^58^. And activation of Wnt/β-catenin signaling led to apoptosis of IVD cells and increase of ECM degradation enzymes, thereby accelerating matrix degradation and promoting IVDD progression^52,59^. However, the relationship between LSI surgery and Wnt/β-catenin signaling in IVDD mice is largely elusive. Our results found that LSI administration up-regulated Wnt/β-catenin signaling, accompanied by increased Adamts5 and Mmp3 in IVDs. Recent evidence also indicated that down-regulating Wnt/β-catenin signaling could suppress Nlrp3 inflammasome activation^60^. Consistent with the above findings, we found that the activation of Wnt/β-catenin signaling preceded the increase of Nlrp3, Caspase-1 and IL-1β in IVD tissues of IVDD mice. These findings indicated that LSI treatment might promote pyroptosis via activation of Wnt signaling.

As stated above, one limitation of this study is that not all aspects of pathological factors of IVDD can be taken into consideration in experimental animal models, and the experimental animal model can only partially resemble complex human conditions. However, this simplified model is extremely useful in investigating the reciprocal interactions between mechanical loading and IVDD progression. In this study, there was a significant induction effect on pyroptosis and innervation of IVDs in IVDD mice, which suggested that the therapy of anti-pyroptosis and anti-innervation may have therapeutic potential for clinical IVDD. However, whether the therapy was able to directly target the intervertebral disc, as well as serious side effects on the human body is still masked. In addition, *Col2a1-CreER*^*T2*^ transgenic mice cannot effectively target all disc cells, especially NP cells. We have constructed *Aggrecan-CreER*^*T2*^ transgenic mice to specifically target disc cells^61^, which enable us to further explore the relationships between Wnt/β-catenin signaling and mechanical stress, pyroptosis and nerve ingrowth during the pathological process of IVDD.

To sum up, we found a mouse IVDD modeling by operating the LSI surgery. Aberrant mechanical stress-induced lumbar IVDD model may expand our knowledge regarding the occurrence and development of IVDD, and also may help explain why osteophytes and spinal stenosis occur. Simultaneously, the study reminded us that restoring the normal mechanical environment of IVD, mitigating IVD pyroptosis, or inhibiting Wnt/β-catenin signaling pathway could have therapeutic potential for clinical IVDD.

## Methods

### Experimental animals

A total of 48 C57BL/6 J male mice (8-week-old, 22 ± 2 g) were purchased from Shanghai Laboratory Animal Center and housed at the Animal Care Facility of Chinese Medical University according to the institutional guidelines for laboratory animals. The ARRIVE (Animals in Research: Reporting In Vivo Experiments) guidelines for reporting animal research were carried out^62^. All the animal procedures were approved by the Ethical Committee of Zhejiang Chinese Medical University (IACUC-20190930–03).

### Surgical procedure

All mice were randomly divided into the LSI model group and the Sham group (n = 24 per group). After being anaesthetized with sodium pentobarbital, mice were placed on the surgical table in a prone position. The L5 vertebra was located on the anterior superior iliac spine. The lower dorsal skin was shaved and disinfected using iodine solution, then a longitudinal incision was created along the midline of the back to expose the lower lumbar spine. The posterior paravertebral muscles from the L3–L5 vertebrae were separated to expose the lumbar 3rd–5th spinous processes, which were further resected along with the supraspinous and interspinous ligaments using a fine scissor. Mice in the Sham group only received a detachment of the posterior paravertebral muscles from the L3–L5 vertebrae (Fig. 1b). After the surgery, the muscles and skin were successively sutured to allow the mice to recover. L3-L5 vertebrae of mice were harvested 1-, 2-, 4-, and 8-week after LSI surgery (Fig. 1a) (n = 6 per group at indicated times).

### μCT analysis

Before histological processing, we firstly evaluated paraformaldehyde-fixed vertebrae by μCT analysis as we previously described^63^. By using a high-resolution μCT (Skyscan 1176; Bruker μCT, Kontich, Belgium) with 90 kVp source and 300 μA current, we scanned vertebrae at a resolution of 9 μm. Three-dimension model visualization software, CTVol v2.0 (Skyscan company, San Jose, CA, USA), was employed to analyze parameters of the L4-L5 IVD with half-height of the L4 and L5 vertebrae. To quantify disc space, we measured the distance between L4 and L5 from μCT images by Image Pro Plus 4.5 (Media Cybernetics, Silver Spring, USA). L5 vertebral body TV was described to figure out the medial compartment excluding cortical bone, transverse and spinous processes. Three-dimensional structural parameters analyzed included: BS/TV, Tb.N, Tb.Sp.

### Histology, IHC and IF analysis

The tissues were fixed in 4% buffered paraformaldehyde for 72 h, decalcified in 14% EDTA (pH 7.4) for 3 weeks at room temperature, dehydrated and then embedded in paraffin. The lumbar tissues were processed into 5-mm-thick coronal-oriented sections for Safranin O/Fast green staining. Briefly, sections were incubated with primary antibodies to Aggrecan (1:300, Abcam, Cambridge, UK), Col2 (1:1000, NeoMarkers), Mmp3 (1:300, Ruiying biological, Suzhou, China), Adamts5 (1:300, Abcam, Cambridge, UK), Nlrp3 (1:800, Proteintech, Wuhan, China), Caspase-1 (1:300, Proteintech, Wuhan, China), IL-1β (1:800, Bioss, Woburn, MA, USA), Cgrp (1:200, Abcam, Cambridge, UK), β-catenin (1:500, Ruiying biological, Suzhou, China), Tcf4 (1:300, Ruiying biological, Suzhou, China), Dkk1 (1:200, Ruiying biological, Suzhou, China) and Ccn2 (1:200, CWBIO, Beijing, China) at 4 °C overnight. For IHC staining, a horseradish peroxidase streptavidin detection system (ZSGB-BIO, Beijing, China) was subsequently used to detect the immunoactivity, followed by counterstaining with hematoxylin (Sigma-Aldrich, St. Louis, MO, USA). For IF analysis, the slides were incubated with secondary antibodies conjugated with fluorescence at room temperature for 1 h. The morphometric study was performed using the Image Analysis System (Olympus, Japan). Triplicates of each sample were used for staining. Quantitative histomorphometric analysis was conducted in a blinded manner with Image-Pro Plus Software version 6.0 (Media Cybernetics Inc, Rockville, Maryland, USA) as we previously described^63^. Histological score was graded in a blind fashion using the definition established by Norcross et al.^28^, with some modifications (Table 1).

**Table 1:** Histological grading scale criteria based on Norcross et al.^28^.

Scores	Nucleus pulposus (NP) symptoms
5	Large, bulging central cavity with abundant NP material; > 2/3 (IVD) height; smooth borders with minimal disruption
4	Slightly reduced central cavity size with some NP material present; > 1/3 IVD height and < 2/3 IVD height; minimal border disruption may be present
3	Markedly reduced and disrupted cavity with minimal NP material and compartmentalization; total cavity > 1/3 IVD height and < 2/3 IVD height
2	Severe disruption of NP with minimal cavity; total cavity < 1/3 IVD height but > 0; consists only of a few small pockets lined by NP-like cells
1	Complete obliteration of cavity with no NP-lined pockets
**Scores**	**Annulus fibrosus (AF) symptoms**
5	Discrete, well-opposed lamellae bulging outward with no infolding; minimal preparation defect with “simple radial clefting”
4	Discrete lamellae, less well-opposed; minimal infolding may be present; fibers remain well-organized, but with ‘‘complex radial clefting’’
3	Moderate to severe infolding of discrete, relatively well-opposed lamellae; moderate fragmentation of lamellae; AF fibers remain well organized
2	Severe infolding and distortion of poorly opposed lamellae; severe fragmentation of lamellae; small regions of disorganized fibrous material replacing central lamellae
1	Severe infolding, distortion, and fragmentation of lamellae; extensive amount of disorganized fibrous material replacing central lamellae
**Scores**	**Cartilage endplate (EP) symptoms**
5	Neatly arranged chondrocytes without any lesion or ectopic bone formation
4	Hyaline cartilage cells were replaced by the spindle-shaped chondrocytes, ectopic bone formation only located at the “junction”
3	Ectopic bone formation located at superior cartilage endplate with smooth cartilage surface
2	Ectopic bone formation located at whole cartilage endplate with smooth cartilage surface
1	Ectopic bone formation located at whole cartilage endplate with flawed cartilage surface

This scale mainly scores the disruption of nucleus pulposus central cavity, cellularity and collagen fiber orientation of annulus fibrosus and the degree of ectopia ossification of cartilage endplate. Simple radial clefting = the presence of radial gaps between AF lamellae with minimal fragmentation; complex radial clefting = the presence of radial, transverse, and/or oblique gaps in the lamellae with significant fragmentation; junction = the triangle junction between the cartilage endplate and the annulus fibrosus.

### TUNEL assay

The TUNEL assay for detecting DNA breaks was performed with TUNEL Bright Green Apoptosis Detection Kit according to the manufacturer’s instruction (Vazyme Biotech; Nanjing, China) as we previously described^63^. Negative controls were incubated in a TdT free-enzyme solution. The number of positive cells was quantified in three randomly selected fields of view using three sections from each sample. DAPI staining was used to estimate the total cell number.

### Real-time quantitative PCR analysis

Total RNA was extracted from L4-L5 IVD using TRIzol reagent (Invitrogen, Carlsbad, CA, USA) following the manufacturer’s protocol. The yield and purity of RNA were quantified using a Nanodrop 2000C spectrophotometer (Thermo Fisher Scientific, Massachusetts, USA) and 2 μg of RNA were reverse-transcribed into cDNA using the Prime Script RT Reagent Kit protocol (Takara Bio, Dalian, China). Real-time PCR amplification was performed using murine gene-specific primers and TB Green Premix Ex Taq II (Takara Bio, Dalian, China). The PCR program was: initial 2 min at 95 °C denaturing; 10 s at 95 °C denaturing; and 30 s annealing/elongation for a total of 40 cycles. The primers used for the PCR are listed in Table 2 and the gel electrophoresis was done to make sure the primers were available. The data were analyzed using the 2^-ΔΔCT^ method^64^.

**Table 2:** Primers used for quantitative RT-PCR.

Genes	Primers (5–3′)	Products (bp)
*β-actin*	S: CACGATGGAGGGGCCGGACTCATC AS: TAAAGACCTCTATGCCAACACAGT	252
*Aggrecan*	S: CCTGCTACTTCATCGACCCC AS: AGATGCTGTTGACTCGAACCT	150
*β-catenin*	S: ATGGAGCCGGACAGAAAAGC AS: CTTGCCACTCAGGGAAGGA	108
*Lef1*	S: CCTCCTACTCCAGTTACTCT AS: GGCACTTTATTTGATGTCCTC	112

### Statistics analysis

All the data were expressed as mean ± standard error of mean (sem), as indicated in the figure legends. Statistical significance was determined using Student’s *t*-test. All data analysis was performed using SPSS 15.0 analysis software (SPSS Inc, Chicago, Illinois, USA) and the level of significance was defined as *P* < 0.05.
